# Open-Healing Socket Preservation with a Novel Dense Polytetrafluoroethylene (dPTFE) Membrane: A Retrospective Clinical Study

**DOI:** 10.3390/medicina56050216

**Published:** 2020-04-28

**Authors:** Gregor-Georg Zafiropoulos, Zeljka Perić Kačarević, Syed Saad B. Qasim, Branko Trajkovski

**Affiliations:** 1 Faculty of Dentistry, Kuwait University, Safat 13110, Kuwait; 2 Department of Anatomy Histology, Embryology, Pathology Anatomy and Pathology Histology, Faculty of Dental Medicine and Health, University of Osijek, 31000 Osijek, Croatia; 3 Wound Healing and Oral Diagnostic Research Group, College of Dental Medicine, University of Sharjah, Sharjah 27272, UAE

**Keywords:** socket preservation, immediate dental implant, open healing, ridge preservation, guided tissue regeneration, PTFE membrane

## Abstract

*Background and objectives:* Non-resorbable dense polytetrafluoroethylene (dPTFE) membranes are widely used for regeneration procedures, alone or in combination with particulate materials. The aim of this work was to examine the efficacy of a newly developed dPTFE membrane in the management of extraction socket healing. *Materials and Methods:* The extraction premolar sockets of 44 patients (20 men and 24 women) were preserved. One group received prosthetic rehabilitation with a fixed partial denture (FPD) (PROS group, N = 19) and a second group received immediate implant placement (IMPL group, N = 25). The PROS group sockets were augmented with a bovine derived xenograft and covered with a newly developed dPTFE membrane prior to FPD rehabilitation. *Results:* In the IMPL group, socket preservation was combined with immediate implant placement. Before (T0) and 6 months after surgery (T1), horizontal and vertical dimensions were measured with customized stents. No significant differences in alveolar bone loss from T0 to T1 were observed between the PROS and IMPL groups in the horizontal dimension for any tooth type. There was a significant difference in alveolar bone loss from T0 to T1 between the two groups for only single-rooted maxillary premolars in the vertical dimension. *Conclusions:* The use of the examined new dPTFE membrane consistently led to the preservation of hard tissue in the extraction sites.

## 1. Introduction

Alveolar bone atrophy following tooth extraction can be a contraindication for dental implant placement or impair the esthetic outcome of a placed implant or conversional prosthetic restoration. Approximately 40% of alveolar height and 60% of alveolar width are susceptible to alveolar bone resorption within 6 months of tooth extraction [1,2]. This resorption increases the risk of buccal soft tissue recession, and the associated interdental bone loss can alter interdental gingival structure [3,4,5].

In the attempt to reduce the need for advanced surgical procedures and to simplify the treatment plan, several surgical techniques were developed to reduce post-extractive alveolar atrophy [6]. Socket preservation (SP), i.e., alveolar ridge preservation, with the application of different biomaterials, is the most common procedure aiming to control crestal bone resorption following dental extractions [7,8]. Over the past two decades, the goal has not been only to solve the existing problem of bone atrophy following tooth extraction, but mainly to avoid the problem or at least to reduce the amount of reabsorption over time. Several different techniques and approaches offer clinicians different possibilities to reach this goal. For this reason, a variety of SP treatment modalities have been described, including socket grafting with a biomaterial alone, the overbuilding of the facial bone wall, occluding access to the socket by interposing a barrier element, or a combination of some of them, with or without using soft tissue grafts to allow primary intention healing [9]. The results of a recent systematic review showed that SP results in a significant reduction in the vertical bone dimensional change following tooth extraction when compared to unassisted socket healing. The reduction in horizontal alveolar bone dimensional change was found to be variable. No evidence was identified to clearly indicate the superior impact of any type of SP procedure (GBR, socket filler and socket seal) on bone dimensional preservation, bone formation and patient complications [10].

Extraction socket healing management options have been improved by the availability of non-resorbable membrane barriers for guided tissue regeneration procedures; such barriers can be used alone or in combination with particulate materials [5]. The application of such membranes is intended to create a volume-space that facilitates the formation of a blood clot, which then serves as a matrix for bone formation, thereby supporting bony regeneration. The most commonly used non-resorbable membranes in guided tissue regeneration operations are expanded polytetrafluoroethylene and dense polytetrafluoroethylene (dPTFE) [11,12,13,14,15,16]. Although both membrane types are made of polytetrafluoroethylene, the larger pore size of the former (5–30 µm) enables it to be stretched more than dPTFE (pore size, 0.2 µm) [11,16,17,18]. Socket grafting with dPTFE membranes has been demonstrated to provide excellent results with open healing in animal and clinical investigations [5,19,20,21].

Implant-supported restorations have become a routine treatment. The introduction of clinical protocols for early, or even immediate, implant loading and placement can provide patients with short treatment durations for implant-supported restorations [22,23,24,25]. Unfortunately, variable implant survival rates have been observed, likely due to unpredictable membrane behavior during open healing. It is hoped that new dPTFE membrane products may improve membrane behavior and thus increase good implant survival outcomes [26]. The aim of the present study was to determine the effectiveness of a novel dPTFE membrane in preserving the dimensions of extraction sockets, with or without immediate implant placement, during a 6-month observation period.

## 2. Materials and Methods

### 2.1. Study Population

Single sockets of 44 extracted teeth in 44 patients (20 men and 24 women; mean age, 48.02 years; age range (mean ± standard deviation): 26–74 years), scheduled for socket preservation, were evaluated in this private practice-based, non-randomized retrospective study. All patients were non-smokers, had a history of periodontitis, and had received scaling/root planning periodontal treatment at least 2 months prior to the surgery, by the same periodontist who performed the surgical treatment described in the present report. All 44 patients had good oral hygiene and compliance: a mean bleeding on probing of 6%; and a mean plaque index of 10%.

The patients were informed about the treatment procedures and given at least 2 weeks to consider their treatment options before an informed consent form was signed. The scheduled treatment procedures were approved by local authorities in Germany before patients provided informed consent. Two treatment groups were formed based on the patients’ treatment decisions. One group received socket preservation following by prosthetic rehabilitation of the edentulous area with a fixed partial denture (FPD) (PROS group, N = 19; 6 men and 12 women; mean age, 45.05 years; age range (mean ± SD), 32–65 years). In the second group, socket preservation was combined with immediate implant placement (IMPL group, N = 25; 13 men and 12 women; mean age, 50.88 years; age range (mean ± SD), 26–74 years). 

The following inclusion criteria were applied: (1) no contraindications for treatment (i.e., systemic disease, pregnancy, the use of prescription or recreational drugs, or being a smoker); (2) only 1 tooth to be extracted, with teeth adjacent to the extraction socket free of caries or previously restored; (3) extraction indicated by mobility, severe dental caries, root caries, fracture, endodontically non-salvageable complications, or rejection of endodontic treatment by the patient; and (4) buccal and lingual/palatal plate of the extraction socket present. Patients were excluded from this retrospective study if their surgery involved all molars, if maxillary premolar areas had insufficient bone height necessitating sinus augmentation prior to implant placement, or if the most distal tooth in the quadrant was missing buccal bone wall.

### 2.2. Surgical Procedure

All surgeries were performed by the same surgeon using the technique described by Bartee [20]. At the time of surgery (T0), an intrasulcular incision was made extending to adjacent teeth, and a full thickness flap was mobilized with horizontal periosteal slitting in the most apical part. No vertical releasing incisions were made. Extractions were performed using an atraumatic technique. The socket was curetted carefully and irrigated with sterile saline solution. Following irrigation, the status of the buccal wall was confirmed by direct examination.

**Figure 1:**
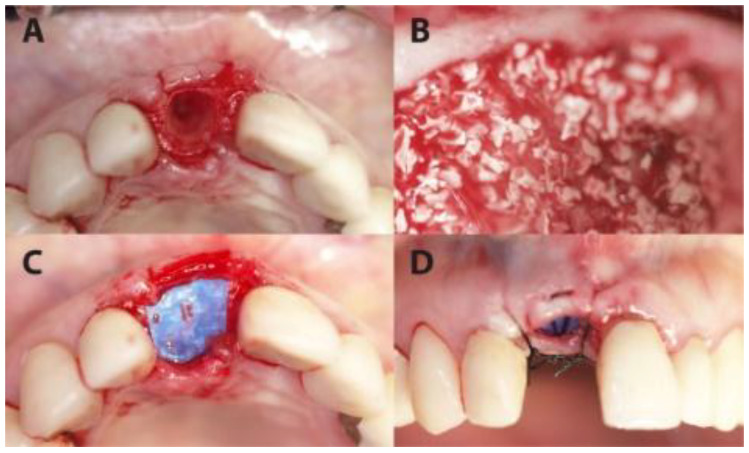
A case from the PROS group in which dental site #11 was treated. (**A**) The extraction socket. (**B**) The socket filled up with xenograft. (**C**) The coverage of the socket with permamem^®^ membrane. (**D**) Flap suturing, leaving the membrane partially exposed.

**Figure 2:**
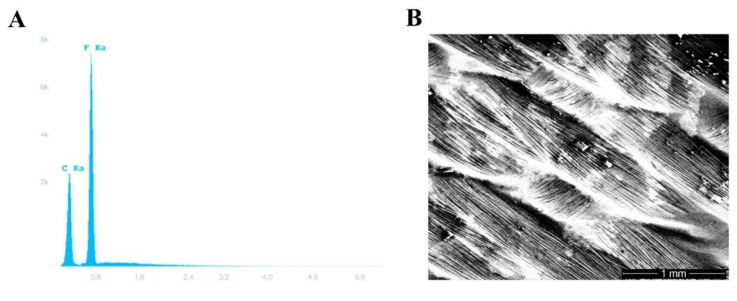
Polytetrafluoroethylene (PTFE) membrane testing. (**A**) Energy-dispersive X-ray spectroscopy showing the characteristic carbon and fluorine peaks of permamem^®^. (**B**) A scanning electron photomicrograph of permamem^®^ (magnification 31×).

Socket preservation was performed with sintered bovine xenograft (cerabone^®^, botiss biomaterials GmbH, Zossen, Germany). Each preserved socket was covered with ultra-thin (~0.08 mm) dPTFE membrane (permamem^®^, botiss biomaterials GmbH, Zossen, Germany) as shown in Figure 1 and Figure 2. 

**Figure 3:**
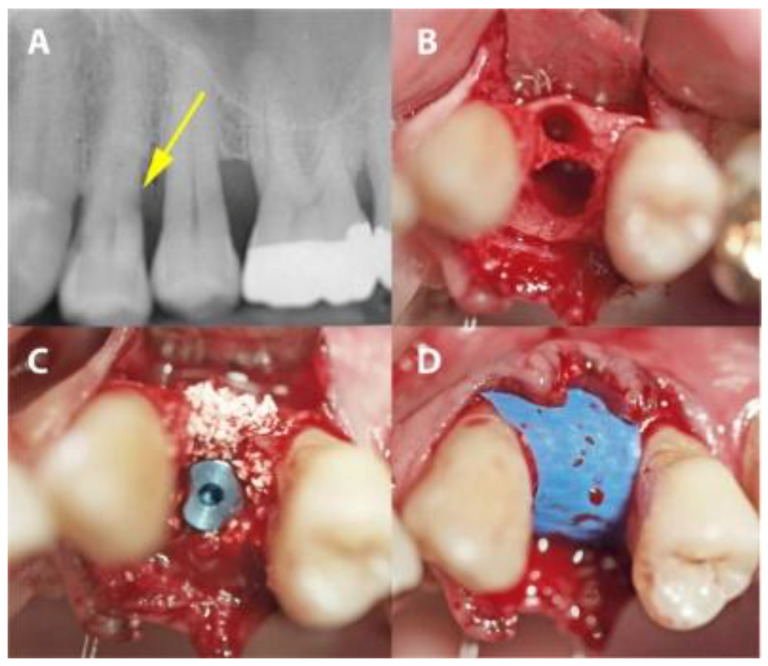
A case from the IMPL group in which dental site #24 was treated. (**A**) A radiograph showing distal subgingival root caries. (**B**) The extraction socket. (**C**) Implant placement with xenographic bone graft augmentation of the gap. (**D**) The PTFE-covered implant site.

In the PROS group, the sockets were preserved as described above. In the IMPL group, a two-stage protocol was applied. Firstly, implants (Bone Level implants, Institute Straumann, Basel, Switzerland) were placed so that the implant body was surrounded by bone. Secondly, gaps around the placed implant were augmented with xenograft material as in the PROS group and then covered with the same dPTFE membrane as in the PROS group (Figure 3).

In both groups, no further steps were taken to secure the membrane in place, and the membrane was left partially exposed during the healing period. The flap was repositioned without tension and sutured to the adjacent papillae with interrupted sutures (Ethibond, Excel 3-0, Johnson & Johnson, Sint-Stevens-Woluwe, Belgium). 

### 2.3. Measurements and Prosthetic Restoration

According to each patient’s choice, the partially edentulous area was either temporarily restored with a removable denture 3 days after surgery or left without temporary restoration. The implants were evaluated 6 months after surgery (T1) according to the criteria of Smith and Zarb [27]. Peri-implant radiolucency, mobility, pain, discomfort, and/or neurosensory alteration were considered indicators of potential implant failure. Hard-tissue evaluation was performed with cone-beam computed tomography (CBCT) before final FPD loading at T1. Buccal plates at all target sites were examined and their presence or resorption was recorded.

Standardized measurements were taken intraoperatively at T0 (immediately after extraction) and at T1. Measurements were performed in the same area of the selected defect at both timepoints. Measurements were made after the removal of any remaining soft tissue from the socket, and before augmentation and membrane placement. Measurements were taken with a caliper and rounded up to the nearest 0.1 mm. The examiner who performed the measurements was not the surgeon.

**Figure 4:**
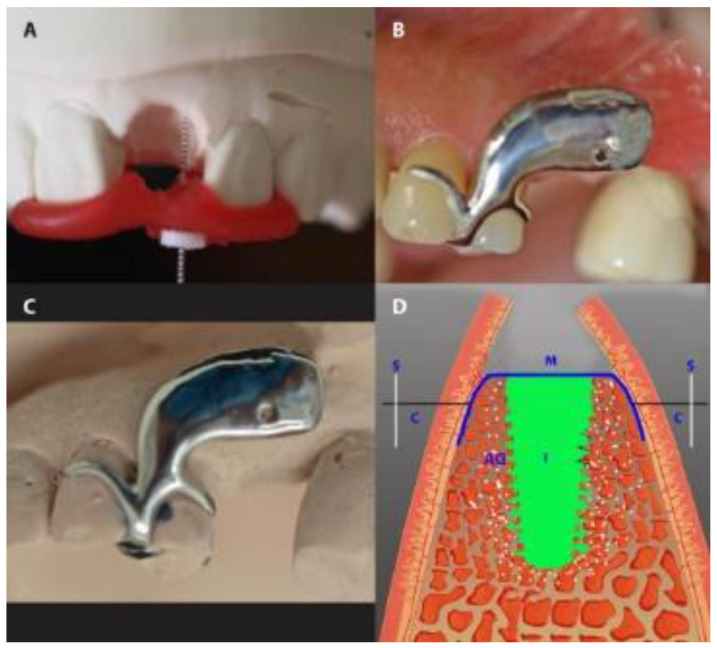
Gap measurement in the IMPL group. (**A**) The measurement of the vertical dimension with an endodontic reamer. (**B**,**C**) The use of a customized stent to measure the buccal-lingual dimension of a socket in a patient’s mouth (**B**) and on the same patient’s dental cast (**C**). (**D**) A diagrammatic presentation of the horizontal measurement. S, stent; M, membrane; I, implant; AG, augmented gap.

The vertical dimension was measured with a stent fabricated in situ during surgery from modeling resin (Pattern Resin^®^; GC, Alsip, IL, USA), immediately after tooth extraction. During polymerization and under light pressure, an endodontic reamer (#15; Dentsply DeTrey, Konstanz, Germany) with a silicone disc stop was positioned perpendicularly to the defect to measure the vertical distances of the mid-mesial and mid-distal socket edges. The disc stop was fixed with cyanoacrylate. After careful removal of the reamer, the vertical distance was measured to the nearest 0.1 mm with a caliper (Figure 4). The mean of the mesial and distal measurements was taken as the mean vertical distance.

**Figure 5:**
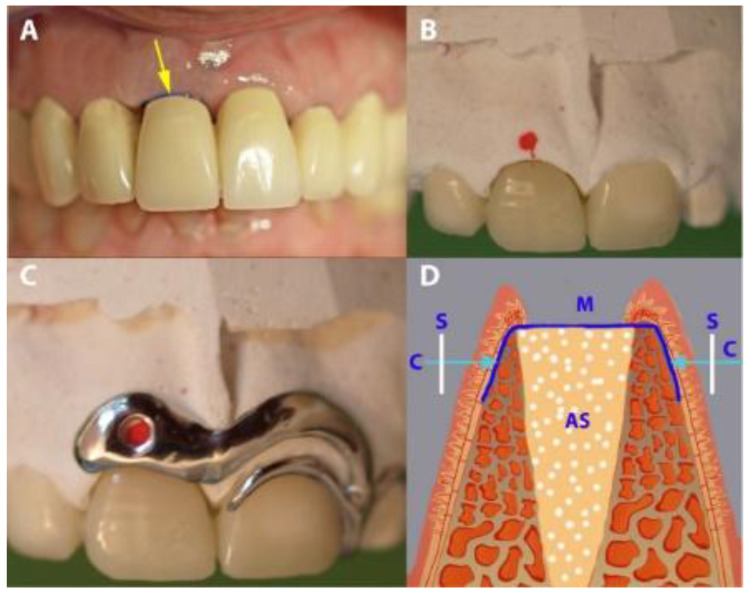
Gap measurement in the PROS group. (**A**) A temporary fixed partial denture (tFPD) in place (the arrow showing the exposed occlusal part of the membrane). (**B**) A tFPD and dental cast, with red points indicating the place of measurement. (**C**) The placement of the stent over the tFPD and dental cast. (**D**) A diagrammatic presentation of the horizontal measurement. S, stent; M, membrane; AS, augmented socket.

In preparation for measuring buccal-palatal/lingual distance (horizontal dimension), in the IMPL group, an impression-based cast was made preoperatively. A customized cobalt-chromium alloy stent (8–10 mm) was fabricated and laid over the operated area immediately after the extraction. The stent had one hole, marking 2.5 mm below the buccal crest of the extraction socket. Using light pressure, the caliper was inserted through the hole, perpendicularly through the mucosal surface to the cortical bone from both the buccal and the palatal/lingual sites (Figure 4). In the PROS group, teeth adjacent to the target tooth were prepared before the operation, and a long-term temporary FPD (tFPD) was milled from Zenotec^®^ colored polymethyl methacrylate (Wieland, Pforzheim, Germany) with an ovate pontic design [28] and margins located in the cemento-enamel junction. Intraoperatively, the tFPD was tested in place and modified to suit the requirements of each patient (i.e., shorted so that it had passive contact with the membrane). The tFPD was fixed on the abutments with TempBond^®^ adhesive (Kerr, Orange, CA, USA). The stent was mounted over the tFPD and buccal-palatal/lingual distance measurements were taken as described above (Figure 5). In both groups, final restorations were performed 6 months after the initial operation, at which time measurements were repeated by placing the stent in the same position and applying the same caliper to probe the surrounding bone under local anesthesia.

### 2.4. Medication and Postoperative Care

**Figure 6:**
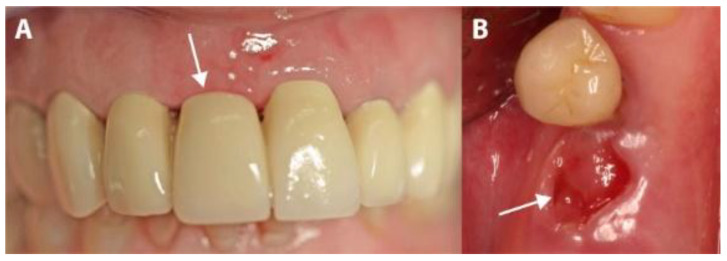
The clinical situation after membrane removal. (**A**) A representative example from the PROS group. (**B**) A representative example from the IMPL group. The arrows indicate non-epithelialized areas.

Patients were prescribed an analgesic (Voltaren^®^ 100 mg, once a day, for 4 days; Novartis Pharma, Nuremberg, Germany) and a systemic antibiotic (Clyndamycin 600 mg, once a day, for 6 days; Ratiopharm, Ulm/Donautal, Germany). They were instructed to start their medication one day before the operation. All patients rinsed twice daily with 0.1% chlorhexidine gluconate solution (GlaxoSmithKline, Buehl, Germany), starting 1 day before the operation and continuing until one week after membrane-removal. Sutures were left in for 8 days, and then the membrane was left for 4 weeks and removed in the fifth postsurgical week after the operation (Figure 6).

During the first eight postoperative weeks, patients underwent weekly maintenance therapy with a dental hygienist. Subsequently, patients were enrolled in supportive periodontal therapy consisting of monthly recall appointments. During the recall appointments, oral hygiene instructions were given, supragingival debridement was performed, chlorhexidine-stains were removed, and teeth were polished.

### 2.5. Prosthetic Restoration

In the PROS group, 6 weeks after membrane removal, final restorations fabricated with a chromium-cobalt alloy (Zenotec NP, Wieland, Pforzheim, Germany) and porcelain veneer (Vintage MP; Shofu, Ratingen, Germany) were fixed with permanent zinc phosphate cement (Harvard Dental, Hoppegarten, Germany). In the IMPL group, 4 months after membrane removal, implants were uncovered and the buccal bone wall was checked with CBCT, and then restored with metal-ceramic crowns (with the same materials as in the PROS group).

### 2.6. Data Analysis

Statistical analysis was performed in the SPSS version 21.0 software (IBM Corp., Armonk, NY, USA). The means and SDs were calculated from the recorded data. A post hoc power analysis was performed. Paired-sample t-tests were used to detect inter-group and inter-timepoint differences. The cut-off for statistical significance was set at *p* < 0.05.

## 3. Results 

Augmentations involved three main sites: maxillary anterior teeth (incisors and/or canines), maxillary premolars (one- and two-rooted) and mandibular premolars. The distributions of treated teeth (N, %) overall and by group, and the demographic characteristics of the patients overall, by group, and by tooth treated are reported in Table 1. During postoperative maintenance phase examinations, none of the patients had bleeding on probing responses exceeding 8%.

Although membranes were left partially exposed during the healing period, none of the 44 patients in this study reported any strong pain and no abscesses, exudates, swelling, allergic reactions or membrane losses were observed during the healing period. Minute amounts of plaque were observed on the exposed surfaces of the membranes. After the membranes were removed in the fifth week (mean time: 30.52 days; range: 27–35 days), non-epithelialized soft tissue was observed in the areas that had been covered by the partially exposed membranes (Figure 6a,b). These tissues re-epithelialized completely in the following 3 to 4 weeks (mean time: 25.02 days; range: 22–29 days). A slight, distinguishable difference in the color of the tissue compared to that of the adjacent tissues remained, and whole keratinized gingiva was preserved. 

**Figure 7:**
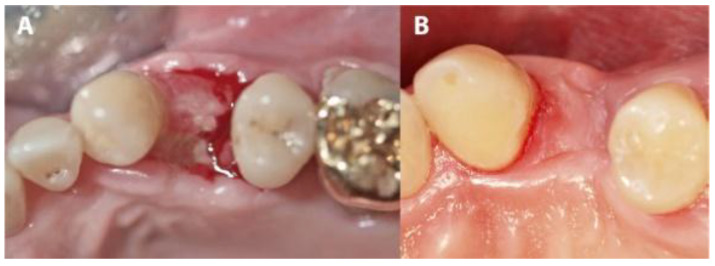
The evolution of a case complicated by suture loss. (**A**) The clinical situation after membrane removal. (**B**) The re-epithelialization of the area shown in panel A, 4 weeks later.

**Figure 8:**
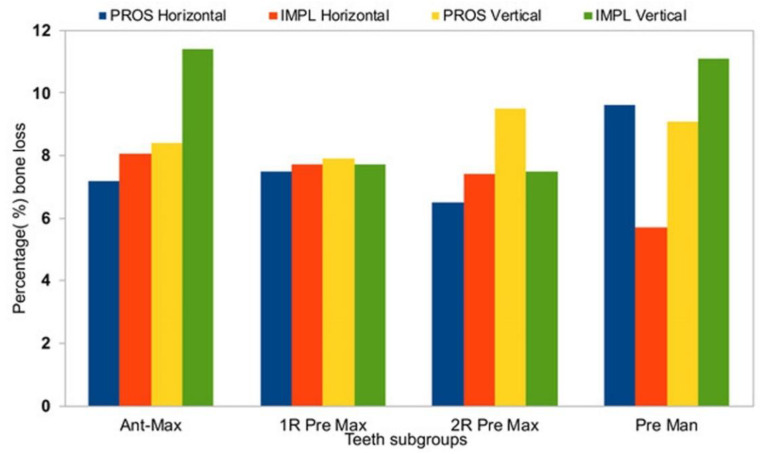
A summary of percentage alveolar bone loss by treatment type group and tooth type subgroup. Ant max, anterior maxilla; 1R Pre Max, 1-rooted maxillary premolars; 2R Pre Max, 2-rooted maxillary premolars; Pre Man, mandibular premolars.

In four IMPL group cases, sutures were lost during the first 3 weeks due to use of an electric toothbrush. Although the membranes remained in place, large areas of the membranes became exposed. In these cases, exposures were mainly observed on the buccal, mesial and distal sites. After membrane removal, non-epithelialized areas were observed where the membranes had been exposed. Re-epithelialization completed during the following 3 to 4 weeks of the healing period (Figure 7 and Figure 8). The complete closure of the sockets with cortical bone was confirmed during implant uncovering.

Horizontal measurements were similar between T0 and T1 for both the PROS and the IMPL groups, demonstrating a significant bone resorption (Table 2, *p* < 0.05), whereas vertical measurements did not show significant losses in alveolar height between T1 and T2 (Table 2). In the PROS group, the range of bone loss in the horizontal dimension was 0.3–0.7 mm (7.2%–9.6%) and the range of bone loss in the vertical dimension was 0.8–1.3 mm (7.9%–9.5%). In the IMPL group, the range of bone loss in the horizontal dimension was 0.3–0.7 mm (5.7%–8.6%) and the range of bone loss in the vertical dimension was 1.0–1.5 mm (7.5%–11.1%). Average changes in bone loss in both dimensions are reported by tooth type in Table 3. In the horizontal dimension, no significant differences in bone loss from T0 to T1 were observed between the PROS and IMPL groups for any of the subtype tooth groups (Table 3). In the vertical dimension, we observed a difference in bone loss from T0 to T1 between the PROS and IMPL groups for the single-rooted maxillary premolars, which was on the edge of significant consideration, perhaps due to the limited patient number (*p* = 0.05; Table 3).

## 4. Discussion

The present private practice-based non-randomized retrospective study demonstrated that the use of a newly developed dPTFE membrane yielded clinically satisfactory regeneration of sockets following tooth extraction. The membranes stayed in place without signs of inflammation or allergic reactions during the open healing period. These findings are in agreement with the outcomes of a recent in vivo preclinical study, in which the biocompatibility of the dPTFE membrane used in this study was affirmed with respect to inflammatory and macrophagic responses [26].

Prior to undergoing their operations, all patients in the present study received an initial periodontal treatment, had good oral hygiene, and were following a strict maintenance regime to ensure the absence of active periodontal disease. No further periodontal records were taken and no adverse tissue reactions or inflammatory changes were observed during the follow-up phase. These positive outcomes may be related, at least in part, to the use of a low-porosity dPTFE membrane that prevents cell adhesion and bacterial penetration [5]. Biofilm management and infection control are essential after periodontal and implant surgery [29]. In this context, chlorhexidine (CHX) mouth-rinses are frequently recommended post-surgically. CHX rinsing helps to reduce biofilm formation and gingival inflammation after surgery. Despite its common use and many studies in this field, a systematic evaluation of CHX’s benefits after periodontal or implant surgery is—surprisingly—still missing [29,30]. Systematic literature searches were performed for clinical trials, which compared CHX rinsing after periodontal or implant surgery with rinsing using placebo, non-staining formulations, or solutions with reduced concentrations of the active compound [29]. The overall tenor of the actual reviews was that CHX may represent a valuable chemo-preventive tool immediately after surgery, during the time period in which oral hygiene capacity is compromised [29,30].

In all cases, there was no primary coverage over the membrane and positive treatment outcomes were observed, consistent with previous studies in which dPTFE membranes were used [5,19,20,21]. Without primary coverage, there is no need to release vertical incisions of the flap, which facilitates the surgical procedure and enhances the esthetic outcome by leaving the muco-gingival junction intact. In addition, due to their relatively smooth surface, dPTFE membranes can, usually, be removed without an additional surgical procedure [19,20,21]. Several protocols for socket preservation using membranes, grafting materials, or a combination of both have been described. Space-maintaining grafting materials can help produce predictable, positive outcomes and prevent the collapse of the membrane and surrounding tissue into the void of the extraction socket [31].

The small areas of plaque accumulation observed on the exposed surfaces of the tested membranes could possibly be consequent to the relatively smooth surface of this type of membrane. This property is certainly positive from a hygiene point of view but could enable greater membrane exposure after suture removal (especially in large mesial-distal or buccal-palatal distance sockets) due to the lack of membrane attachment to the mucosal flap. The same could happen in cases of suture loss (Figure 7 and Figure 8). In the cases in which we observed such exposure, there were resultant high oral hygiene demands and a temporarily compromise in the esthetics. However, no biological or permanent esthetic complications were seen after membrane removal, with complete re-epithelialization proceeding within 3 to 4 weeks. It is important to trim the membrane to the correct dimensions and adapt the flaps in papilla areas properly without tension to avoid this complication [5].

The presently observed 100% implant survival rate is similar to previously reported rates reported for extraction sockets treated with non-resorbable barriers and implants [32,33]. When Hoffman et al. [19] examined regeneration in extraction sockets treated with dPTFE membranes without graft materials, they observed newly formed tissue at the extraction site, mainly in trabecular bone, with 1–2 mm of bone loss in the direct center of the regenerated socket 1 year after surgery. Notwithstanding, the present data cannot be compared directly with those of Hoffmann et al. because of the use of different measuring devices and methodologies.

The use of space-preserving xenografts in the present study could explain the overall preservation of horizontal and vertical distances in this study. Differences in bone loss between tooth types of treatment procedures could be related to differences in root and socket anatomy. 

The positive outcomes obtained in the present study may be the result of initial coagulum stabilization enabled by the membrane over the initial 4 weeks after extraction. It has been postulated that membrane protection of the socket may prevent minor infectious and limit inflammatory processes, making bone apposition more effective. Additionally, the deposition of more compact bone was achieved by excluding the periosteum from the healing process, thereby reducing remodeling phenomena and subsequent osteolysis within the alveolus [32,33,34,35].

The present results demonstrate that extraction socket augmentation and coverage with non-resorbable dPTFE membranes can be employed to preserve sockets effectively, but does not block alveolar bone resorption completely. Immediate implant placement does not prohibit a small amount of atrophy due to residual gaps between the implant body and socket bone walls. In cases where the mesial and/or distal socket edges are higher than buccal or palatal edges, there may be bone loss in the vertical dimension. This study had two to three notable limitations. Firstly, mesial and palatal measurements were not performed. Secondly, the results may not be generalizable to implant protocols that differ from that used here. Thirdly, number of patients was low.

Although the use of dPTFE membranes supports the preservation of ridge width and height, treatment outcomes depend, to some extent, on the architecture of the local bony walls. Newly formed bone appears to follow the contours of surrounding bony tissue, with no or minimal bone formation extending beyond the existing bony walls, as demonstrated in previous studies [5,19,21]. The high density and small pore size (0.2 µm) of the d-PTFE membrane protects underlying grafts and implants from exposure, potentially eliminating bacterial infiltration into an augmented area [18,35,36]. Because the small pore size of d-PTFE membranes reduces bacterial penetration, they can be left exposed in the oral cavity without subsequent infection [15,16,17,18]. Furthermore, because d-PTFE membranes are not attached to tissues, they can be removed through the mucosa without raising a flap [17,19,21,36].

## 5. Conclusions

Polytetrafluoroethylene (dPTFE) membranes have been used for regenerative treatments for many years, and new ones are constantly being developed. In many cases, their efficacy in the management of extraction socket healing remains to be fully investigated. Therefore, we retrospectively examined the results obtained from 44 patients treated with newly developed dPTFE membrane. Based on the limitations of this study, the results indicate that its use in open-healing socket preservation leads, predictably, to the preservation of the hard tissue in extraction sites when used either in combination with xenograft only, or in combination with immediate implant placement. Additional long-term randomized controlled clinical trials with appropriate patient populations are needed to study the final outcomes, potential complications, vertical regeneration of the buccal bone wall, fenestrations and optimization of surgical procedures.

## Figures and Tables

**Table 1:** The demographic characteristics of patients by tooth treated and treatment group.

Cases	Gender, N (%)		Age	Total, N (%)
	Female	Male	Mean (Range)	
**Treated Cases (Total)**	24 (54.54)	20 (45.45)	48.02 (26–42)	44 (100.0)
Tooth type				
Anterior maxilla	7 (58.33)	5 (41.66)	43.75 (26–63)	12 (28)
1-root premolar maxilla	5 (35.71)	9 (64.28)	47.14 (32–74)	14 (32)
2-root premolar maxilla	4 (44.44)	5 (55.55)	52.22 (37–72)	9 (20)
Premolar mandible	7 (77.77)	2 (22.22)	50.88 (35–65)	9 (20)
Treatment group				
IMPL group (Total)	12 (48.0)	13 (52.0)	50.88 (35–65)	25 (57)
PROS group (Total)	12 (63.15)	6 (31.57)	45.05 (32–65)	19 (43)

**Table 2:** Mean horizontal and vertical socket measurements (±SD, mm) from before (T0) and 6 months after (T1) their operations.

*Group*	Vertical Dimension			Horizontal Dimension		
Subgroup	T0	T1	*p*	T0	T1	*p*
IMPL	7.5 ± 1.6	7.0 ± 1.6	0.121	11.9 ± 2.1	10.6 ± 2.1	0.020 *
Anterior maxilla	6.2 ± 1.6	5.7 ± 0.8	0.157	10.5 ± 1.1	9.3 ± 1.3	0.067
1-root premolar maxilla	7.8 ± 0.7	7.2 ± 0.9	0.074	7.8 ± 0.7	7.2 ± 0.9	0.074
2-root premolar maxilla	9.5 ± 0.8	8.8 ± 0.8	0.111	13.3 ± 1.2	12.3 ± 0.9	0.140
Premolar mandible	5.3 ± 0.5	5.0 ± 0.7	0.211	9.0 ± 0.8	8.0 ± 0.8	0.144
PROS	6.6 ± 1.7	6.1 ± 1.7	0.205	10.5 ± 1.1	9.3 ± 1.4	0.043 *
Anterior maxilla	5.7 ± 0.9	5.3 ± 1.0	0.291	11.2 ± 2.2	10.3 ± 2.2	0.163
1-root premolar maxilla	8.0 ± 0.7	7.4 ± 0.6	0.199	12.7 ± 1.9	11.8 ± 2.0	0.337
2-root premolar maxilla	9.3 ± 0.5	8.7 ± 0.6	0.103	9.5 ± 1.3	8.6 ± 1.3	0.156
Premolar mandible	5.2 ± 0.4	4.7 ± 0.4	0.092	11.0 ± 2.0	10.0 ± 2.1	0.064

* *p* < 0.05, T0 vs. T1 within group.

**Table 3:** Mean changes in horizontal and vertical measurements (±SD, C-mm) and associated percentage changes (%C), from before (T0) to 6 months after (T1) operations by treatment group and tooth subtype.

Dimension	PROS		IMPL		*p*
Tooth Type	C-mm	%C	C-mm	%C	
Horizontal					
Anterior maxilla	−0.3 ± 0.3	7.2	−0.5 ± 0.4	8.06	0.23
1-root premolar maxilla	−0.6 ± 0.5	7.5	−0.6 ± 0.4	7.7	0.5
2-root premolar maxilla	−0.7 ± 0.3	6.5	−0.7 ± 0.4	7.4	0.50
Premolar mandible	−0.5 ± 0.4	9.6	−0.3 ± 0.3	5.7	0.31
Vertical					
Anterior maxilla	−1.3 ± 0.5	8.4	−1.2 ± 0.5	11.4	0.39
1-root premolar maxilla	−1.0 ± 0.4	7.9	−1.5 ± 0.6	7.7	0.05
2-root premolar maxilla	−0.8 ± 0.3	9.5	−1.1 ± 0.6	7.5	0.26
Premolar mandible	−0.9 ± 0.6	9.1	−1.0 ± 0.0	11.1	0.41
